# Short-Term, Equipment-Free High Intensity Interval Training Elicits Significant Improvements in Cardiorespiratory Fitness Irrespective of Supervision in Early Adulthood

**DOI:** 10.3389/fspor.2021.697518

**Published:** 2021-07-26

**Authors:** Tanvir S. Sian, Thomas Inns, Amanda Gates, Brett Doleman, Nima Gharahdaghi, Philip J. Atherton, Jon N. Lund, Bethan E. Phillips

**Affiliations:** ^1^MRC-Versus Arthritis Centre for Musculoskeletal Ageing Research and NIHR Nottingham Biomedical Research Centre, School of Medicine, University of Nottingham, Derby, United Kingdom; ^2^Department of Surgery and Anaesthesia, Royal Derby Hospital, University Hospitals of Derby and Burton, Derby, United Kingdom; ^3^University of Nottingham, Derby, United Kingdom

**Keywords:** high intensity (strenuous) exercise, skeletal muscle, cardiorespiratory fitness, lifestyle and behaviour, health

## Abstract

**Introduction:** Serious health implications from having low levels of cardiorespiratory fitness (CRF) and being overweight in young adulthood are carried forward into later life. High-intensity interval training (HIIT) is a time-effective, potent stimulus for improving CRF and indices of cardiometabolic health. To date, few studies have investigated the use of equipment-free HIIT or the impact of supervision for improving CRF via HIIT.

**Methods:** Thirty healthy young adults (18–30 y) were randomised to 4 weeks (12 sessions) equipment-free, bodyweight based supervised laboratory HIIT (L-HIIT), unsupervised home HIIT (H-HIIT) or no-intervention (CON). Utilised exercises were star jumps, squats and standing sprints. Measurements of CRF (anaerobic threshold (AT) and VO_2_peak), blood pressure (BP), body mass index (BMI), blood glucose and plasma insulin by oral glucose tolerance test (OGTT), and muscle architecture were performed at baseline and after the intervention.

**Results:** When compared to the control group, both HIIT protocols improved CRF (AT: L-HIIT mean difference compared to the control group (MD) +2.1 (95% CI: 0.34–4.03) ml/kg/min; *p* = 0.02; H-HIIT MD +3.01 (1.17–4.85) ml/kg/min; *p* = 0.002), VO_2_peak: L-HIIT (MD +2.94 (0.64–5.25) ml/kg/min; *p* = 0.01; H-HIIT MD +2.55 (0.34–4.76) ml/kg/min; *p* = 0.03), BMI (L-HIIT MD −0.43 (−0.86 to 0.00) kg/m^2^; *p* = 0.05; H-HIIT: MD −0.51 (−0.95 to −0.07) kg/m^2^; *p* = 0.03) and *m. vastus lateralis* pennation angle (L-HIIT MD 0.2 (0.13–0.27)°; *p* < 0.001; H-HIIT MD 0.17 (0.09 to 0.24)°; *p* < 0.001). There was no significant change in BP, blood glucose or plasma insulin in any of the groups.

**Conclusions:** Four weeks time-efficient, equipment-free, bodyweight-based HIIT is able to elicit improvements in CRF irrespective of supervision status. Unsupervised HIIT may be a useful tool for counteracting the rise of sedentary behaviours and consequent cardiometabolic disorders in young adults.

## Introduction

The health benefits of being physically active in adolescence and young adulthood are well-documented. These benefits include enhanced cardiorespiratory and muscular fitness, superior bone and cardiometabolic health and a positive effect on body weight status (Guthold et al., 2020), as well as improved cerebrovascular and cognitive functioning abilities (Hwang et al., 2018). There is also evidence that these health benefits are carried forward into later adult life, with cardiorespiratory fitness (CRF) in young adulthood a modifiable, prognostic marker of long-term mortality and cardiovascular disease (CVD) risk in later life (Guthold et al., 2020). Specifically, higher fitness levels in early adulthood are associated with a lower future risk for all-cause mortality and adverse cardiovascular events (Valle et al., 2015; Shah et al., 2016), as well as being associated with a lower risk for developing cardiometabolic disorders, such as type 2 diabetes (T2DM), by middle-age (Chow et al., 2016).

Sedentary behaviour, defined as any waking activity with an energy expenditure <1.5 METs whilst in a sitting, reclining or lying posture (Tremblay et al., 2017), and physcial inactivity are becoming increasingly prevalent in modern societies based on changes in physical, social, and economic environments, especially for younger adults (Owen et al., 2010; Castro et al., 2018). Worryingly, it is well-established that sedentary behaviours are associated with both cardiovascular and all-cause mortality (Kodama et al., 2009; Ekelund et al., 2016; Young et al., 2016). Further, numerous physiological changes associated with chronic disease risk are seen with physical inactivity, including declines in CRF, insulin resistance, hypertension, and increased adiposity (Booth et al., 2017). Together, combined presentation of the latter three risk factors is often termed the Metabolic Syndrome (MetS) (Kassi et al., 2011), which confers an odds ratio for cardiovascular morbidity and mortality of 3 and 1.8, respectively (Isomaa et al., 2001). Independent of other factors, young people with a high body mass index (BMI) have an increased risk of developing MetS in later life and may already have established hallmarks of the cardiometabolic disease such as elevated blood glucose, increased carotid intima thickness and raised serum cholesterol levels (Magnussen et al., 2010). In addition, sedentary behaviour is associated with an increased risk of T2DM (Wilmot et al., 2012; Patterson et al., 2018), with 29% of T2DM incidence in the UK estimated to be associated with physical inactivity (Patterson et al., 2018).

Although physical inactivity is associated with declines in CRF (Booth et al., 2017), physical activity can mitigate these reductions (Sparling et al., 2015) as well as the aforementioned risks of mortality associated with sedentary behaviour (Ekelund et al., 2016). However, despite these clear benefits, up to 62% of young adults do not meet current physical activity guidelines (Valle et al., 2015), with “lack of time” a chief barrier in younger adults (Fisher et al., 2015). Other commonly reported barriers preventing exercise participation in young adults include a lack of access to equipment and negative body image when exercising in public (Korkiakangas et al., 2011; Scott et al., 2020).

High intensity interval training (HIIT) has emerged as a time-efficient strategy to rapidly improve CRF (Gillen et al., 2014; Weston M. et al., 2014) and indices of cardiometabolic health, such as weight loss and insulin sensitivity, in younger adults (Gibala and Little, 2010; Weston K. S. et al., 2014). However, these favourable adaptations, that have been shown to be achieved in a shorter time-frame than those achieved by traditional aerobic exercise training (Gillen et al., 2014) have, for the most part, been achieved by supervised training employing specialist equipment such as cycle ergometers (Karlsen et al., 2017; Scott et al., 2019). To date, very few studies have investigated the efficacy of home-based HIIT regimes for improving CRF, with those that have been conducted studying middle-aged individuals (Blackwell et al., 2017; Scott et al., 2019) or those with disease (Scott et al., 2019). In addition, of those studies that have compared laboratory and home-based HIIT (Blackwell et al., 2017; Scott et al., 2019), different modalities of exercise were employed in each of these settings, and as such the impact of supervision was not explored.

Reducing sedentary behaviour in younger adults should be considered a key strategy to promote health and well-being across the life-course (Carnethon et al., 2003; Vainshelboim et al., 2019). Given the purported barriers to exercise participation in this particular age-group, and the allied growing interest in optimising the magnitude of adaptation from exercise, whilst minimising time and effort (Fisher et al., 2015; Foster et al., 2015; Shepherd et al., 2015), a home-based HIIT regime that can improve CRF without the need for specialist equipment could be largely beneficial in this space (Fisher et al., 2015). Therefore, we investigated both the efficacy of, and the impact of supervision on, short-term, equipment-free HIIT for improving CRF in young, recreationally active adults. We hypothesised that this HIIT regime would improve the CRF of young adults, irrespective of supervision status.

## Methods

### Subject Characteristics

Thirty recreationally active, non-obese participants aged 18–30 (24(5) years, 15 male), not engaged in a formal exercise regimen (defined as 2 or more sessions per week) were recruited to this study (Table 1), with further exclusion criteria taken from the American Thoracic Society (ATS)/American College of Chest Physicians (ACCP) and the Preoperative Exercise Testing and Training Society (POETTS) (Weisman et al., 2003; Levett et al., 2018) guidelines for exercise testing. After health screening by a medical doctor and provision of informed consent, 10 participants were randomised to each intervention group using computer-generated random permuted blocks of: (i) laboratory (supervised) HIIT (L-HIIT), (ii) home (unsupervised) HIIT (H-HIIT), and (iii) a no-intervention control (CON). Ethical approval for the study was obtained from the University of Nottingham Faculty of Medicine and Health Sciences Research Ethics Committee (C16122016) and the study was registered at clinicaltrials.gov (NCT03473990).

**Table 1:** Participant baseline characteristics.

	**L-HIIT (*n* = 10)**	**H-HIIT (*n* = 10)**	**CON (*n* = 10)**
Age (years)	22 (4)	27 (4)	24 (6)
Male/Female	4/6	5/5	6/4
BMI	25 (4)	26 (4)	25 (4)
AT	14.89 (4.6)	20.27 (5.8)	18.22 (4.2)
VO_2_	31.08 (7.9)	36.06 (4.8)	37.86 (8.8)
SBP	120 (11)	126 (6)	118 (14)

*Data depicts mean (SD). L-HIIT, Laboratory (supervised) high intensity interval training; H-HIIT, Home-based (unsupervised) HIIT; CON, No-intervention control group; BMI, body mass index (kg/m^2^); AT, anaerobic threshold (mg/kg/min); VO_2_, VO_2_peak (mg/kg/min); SBP, systolic blood pressure (mmHg). No significant differences between groups*.

### Assessment Sessions

Assessment sessions were conducted before and 72 h after (to ensure CPET performance was not restricted by fatigue or that cardiometabolic measures were not affected by prior acute exercise) a 4-week intervention period. For each of these sessions' participants were asked to fast from 2200 h the preceding night and to attend the research unit at 0900 h. Measures of height and weight were taken to calculate BMI and resting BP was measured, before a 2-h fasted oral glucose tolerance test (OGTT) [World Health Organisation (WHO), 2006]. Plasma insulin concentrations from the OGTT were measured using an ultrasensitive enzyme linked immunosorbent assay (ELISA) according to manufacturer's instructions (Mercodia AB, Uppsala, Sweden), with blood glucose measured on a near-patient glucose analyser (YSI Life Sciences, Ohio, USA). During the OGTT, muscle architecture [fibre pennation angle (PA), muscle thickness (MT) and fascicle length (FL)] was assessed using B-mode ultrasound (MyLab™50; Esaote, Genoa, Italy) as previously described (Rueden et al., 2017; Franchi et al., 2018).

After provision of a standardised meal and a 30 min rest period, a cardiopulmonary exercise testing (CPET) was performed in accordance with the ATS/ACCP and POETTS No Matches Found guidelines on a cycle ergometer (Lode Corival, Lode BV, Groningen, Netherlands) with continuous heart rate, blood pressure (BP) and pulse oximetry as safety monitoring. Breath-by-breath data was collected using an inline gas analysis system (ZAN 680, nSpire Health, Colorado, USA). Each test began with a 3-min period of observed rest on the cycle ergometer followed by a 2-min warm up of unloaded cycling at a sustained cadence of 55 rpm to allow participant acclimatisation to the ergometer. Participants were encouraged to exercise to volitional exhaustion, with termination criteria as set by ATS/ACCP and POETTS (Weisman et al., 2003; Levett et al., 2018) guidelines. VO_2_peak was defined as the highest VO_2_ attained during the test and was determined by the gas analysis system. Anaerobic threshold (AT) was later defined using a combination of the modified V-slope and ventilatory equivalents (VE) methods by two blinded experienced assessors, with disagreement resolved by consensus (Kaczmarek et al., 2019).

### Interventions

**Figure 1:**
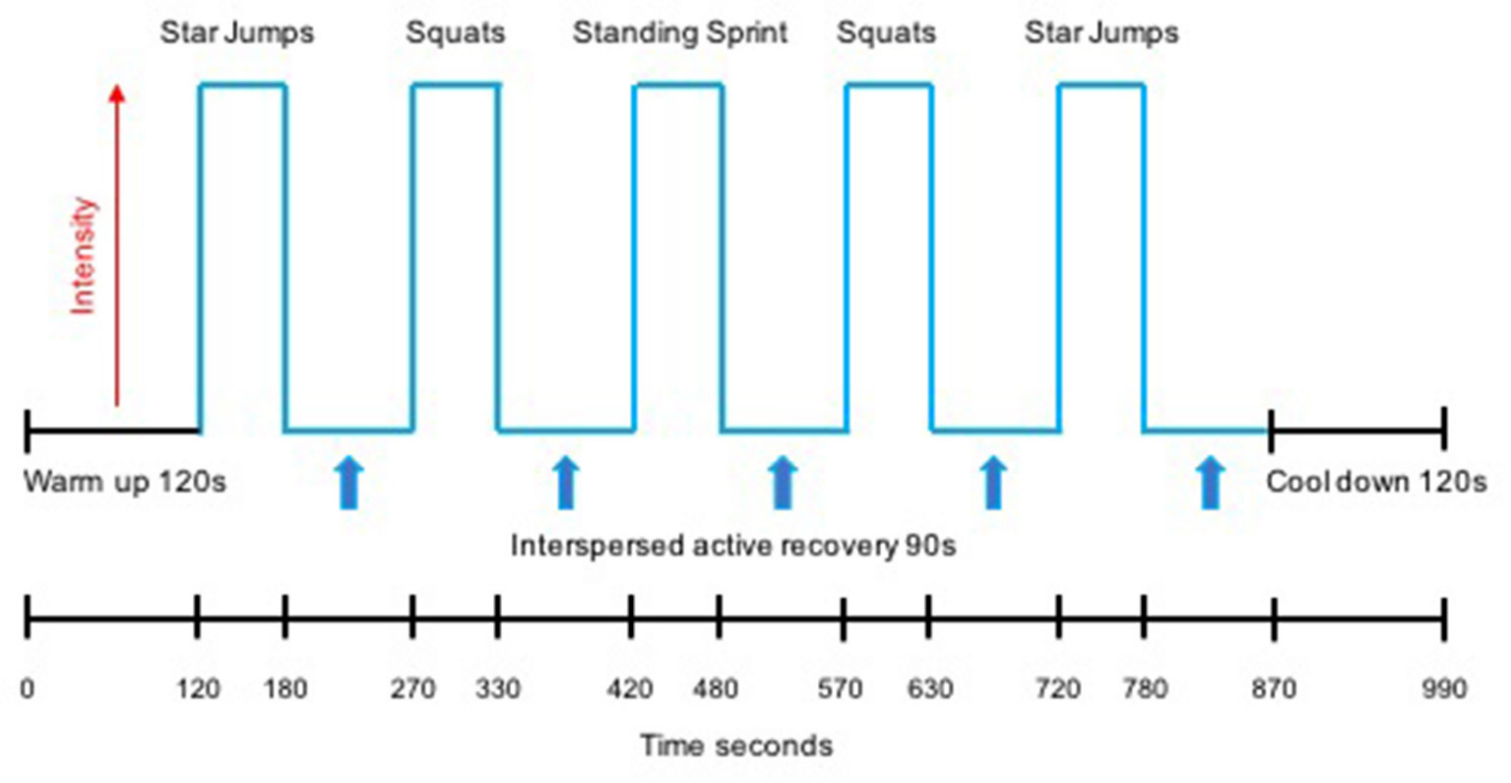
Schematic representation of the high intensity interval training (HIIT) protocol.

The HIIT protocol undertaken by both the L-HIIT and H-HIIT groups was based on previous work demonstrating the efficacy of this temporal protocol to improve CRF when performed on a cycle ergometer (Boereboom et al., 2016; Blackwell et al., 2017) and as bodyweight-based exercises (Blackwell et al., 2017). All sessions began with a warm-up period of 2 min of walking on the spot before five, 1-min high-intensity efforts were performed, each interspersed by 90 s of active recovery. Following the final effort, a 2-min recovery period concluded each session. Exercises were performed in a pyramid design with the same exercise performed for effort 1 as for effort 5 (star jumps) and the same for effort 2 as for effort 4 (standing squats) with the third effort being an on-the-spot (standing) sprint. Participants were instructed to attempt to achieve the same number of repetitions for effort 4 as achieved in effort 2, and for effort 5 as in effort 1. This design aimed to provide motivation to maintain intensity during fatigue (i.e., in efforts 4 and 5) (Figure 1).

All participants assigned to a HIIT group attended the research unit for an in-person introductory session to the HIIT protocol before they started training. This session ensured that all participants were comfortable with the temporal profile of the sessions, were coached for proper form during each exercise, and showed H-HIIT participants how to record engagement with each session. The aim for each effort during every session was to achieve a heart rate >85% age-predicted maximum HR (HR_max_) (Fox et al., 1971), as such constant HR was monitored during this introductory session to allow participants to experience HIIT at the desired intensity.

All participants were asked to maintain their level of physical activity and habitual dietary intake for the duration of the study. Participants in both HIIT groups completed three sessions each week for 4 weeks. L-HIIT participants completed this under one-to-one supervision at the research unit, with verbal encouragement throughout each session. H-HIIT participants completed their sessions at home with no supervision and no direct feedback or communication with the research team. Participants in the H-HIIT group used a pulse oximeter to assess HR at the end of each effort. As with our decision to set our intensity aim at 85% age predicted HR_max_ (rather than true HR_max_ from the initial CPET), pulse oximetry was chosen based on our desire to understand the real-world translational application of this HIIT regime. H-HIIT participants were also given a print resource outlining the HIIT session and specific exercises, and provided information on what to do if they developed any adverse symptoms.

### Statistical Analysis

Based on VO_2_peak data from a previous study by our research group (Blackwell et al., 2017), an *a priori* power calculation determined that 30 participants (10 per group) would be required to achieve an α of 0.05 and a 1–β of 0.80 (de Boer et al., 2015). Descriptive statistics are reported as number (%) or mean (SD). Within group analysis for pre and post intervention change was performed using repeated measures *t*-tests using GraphPad Prism for Mac OS X Version 7.0d (GraphPad Software, La Jolla, San Diego, California USA). Change between groups was determined using ANCOVA with baseline values as the continuous covariates. Effect estimates are presented as mean differences (MD) with 95% confidence intervals (CI). Due to lack of normality, equality of variance and heteroscedasticity for pennation angle analysis, these data were log transformed. Analyses were conducted using Stata Version 16 (StataCorp LLC, TX, USA). The level of significance was set at *p* < 0.05.

## Results

### Study Adherence

After randomisation, one participant in the H-HIIT group did not complete the required number of training sessions (minimum of 10) and as such was excluded from the study. Training adherence (number of sessions completed) and compliance (% of training sessions where the target heart rate threshold of 85% HR_max_ was achieved) was otherwise 100%. There were no adverse safety events reported in any group during exercise testing or training.

### Cardiorespiratory Fitness

**Figure 2:**
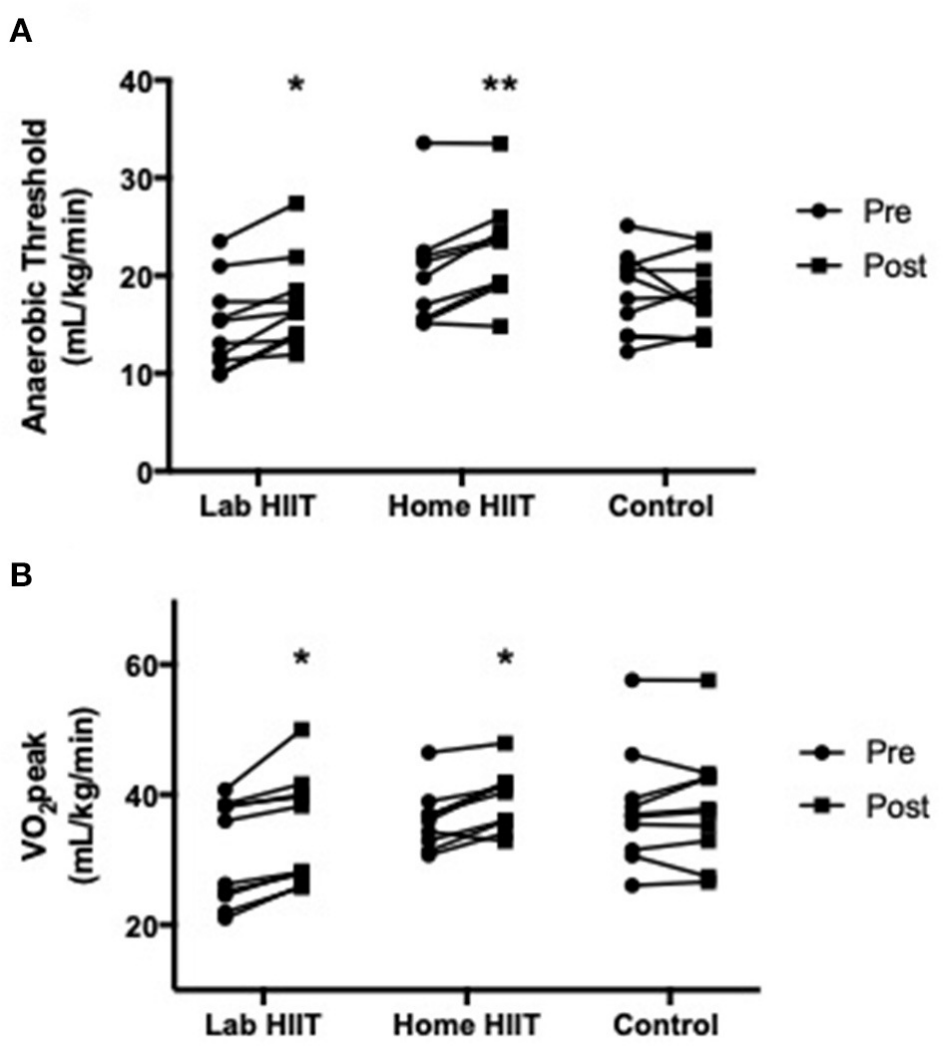
**(A)** Anaerobic threshold and **(B)** VO_2_peak in young individuals before and after lab (*n* = 10) or home-based (*n* = 9) high intensity interval training (HIIT) or a no intervention control (*n* = 10) period. *p < 0.05; **p < 0.01 vs. pre-intervention.

There was a significant increase in AT in the L-HIIT (MD +2.1 (0.34–4.03) ml/kg/min; *p* = 0.02) and H-HIIT groups (MD +3.01 (1.17–4.85) ml/kg/min; *p* = 0.002) after the intervention period compared to control, with no difference in increase between the L-HIIT and H-HIIT groups (MD −0.82 (−2.83 to 1.18) ml/kg/min; *p* = 0.41) (Figure 2A, Supplementary Table 1).

There was a significant increase in VO_2_peak in the L-HIIT (MD +2.94 (0.64–5.25) ml/kg/min; *p* = 0.01) and H-HIIT groups (MD +2.55 (0.34–4.76) ml/kg/min; *p* = 0.03) after the intervention period compared to control. There was no difference in increase between the L-HIIT and H-HIIT groups (MD +0.39 (−1.90 to 2.68) ml/kg/min; *p* = 0.73) (Figure 2B, Supplementary Table 1).

### Resting Blood Pressure

There were no significant changes in SBP in the L-HIIT (MD −1.71 (−6.67 to 3.26 mmHg; *p* = 0.49) or H-HIIT (MD −0.49 (−5.85 to 4.86) mmHg; *p* = 0.85) groups after the intervention period compared to control, with the same findings for DBP (L-HIIT: MD −2.79 (−9.01 to 3.43) mmHg; *p* = 0.37, H-HIIT: MD −0.13 (−6.64 to 6.38) mmHg; *p* = 0.97) (Supplementary Table 1).

### Body Mass Index

**Figure 3:**
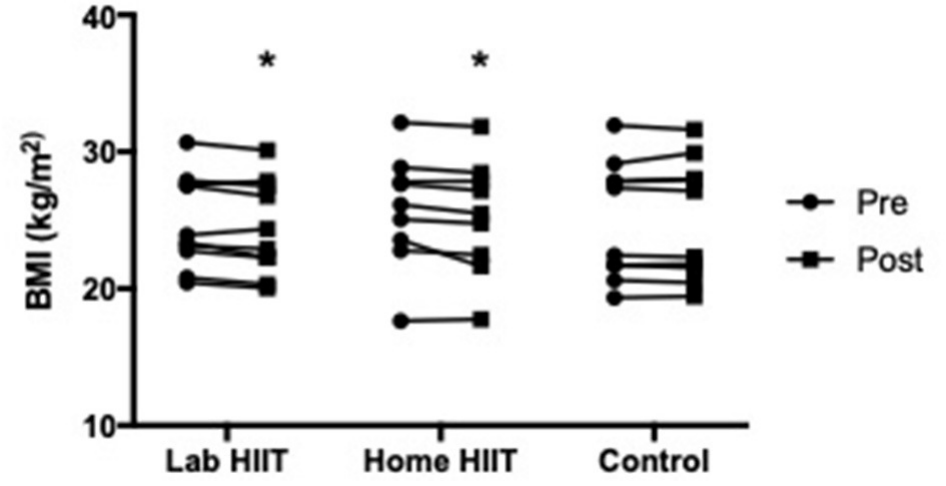
Body mass index (BMI) in young individuals before and after lab (*n* = 10) or home-based (*n* = 9) high intensity interval training (HIIT) or a no intervention control (*n* = 10) period. *p < 0.05 vs. pre-intervention.

There was a significant decrease in BMI in the L-HIIT (MD −0.43 (−0.86 to 0.00) kg/m^2^; *p* = 0.05) and H-HIIT (MD −0.51 (−0.95 to −0.07) kg/m^2^; *p* = 0.03), groups after the intervention period compared to control, with no difference in the magnitude of change between the HIIT groups (MD 0.08 (95% CI −0.37 to 0.52) kg/m^2^; *p* = 0.72) (Figure 3, Supplementary Table 1).

### Muscle Architecture

There was a significant increase in *m. vastus lateralis* PA in the L-HIIT (MD +0.2 (0.13–0.27)°; *p* < 0.001) and H-HIIT groups (MD +0.17 (0.09–0.24)°; *p* < 0.001) after the intervention period compared to control, with no significant difference in increase between the L-HIIT and H-HIIT groups (MD +0.03 (−0.04 to 0.11)°; *p* = 0.36) (Supplementary Table 1).

There were no significant changes in *m. vastus lateralis* MT (L-HIIT: MD +0.03 (−0.13 to 0.19) mm; *p* = 0.72, H-HIIT: MD −0.05 (−0.21 to 0.12) mm; *p* = 0.58) or FL (L-HIIT: MD −0.07 (−0.62 to 0.48) mm; *p* = 0.8, H-HIIT: MD 0.59 (0 to 1.18) mm; *p* = 0.06) in either HIIT group after the intervention period compared to control (Supplementary Table 1).

### Blood Glucose and Plasma Insulin Concentrations

There were no significant changes in blood glucose area-under-the-curve (AUC) for the OGTT in the L-HIIT (MD −58.36 (−175.58 to 58.85) mmol/120 min; *p* = 0.31) or H-HIIT (MD −55.19 (−171.16 to 60.78) mmol/min; *p* = 0.33) groups after the intervention period compared to control (Supplementary Table 1).

Similarly, there were no significant changes in insulin AUC in either HIIT group (L-HIIT: MD +1127.61 (−1445.29 to 3700.51) uU/ml/120 min; *p* = 0.37, H-HIIT: MD +494.3 (−2120.47 to 3109.08) uU/ml/120 min; *p* = 0.7) after the intervention period compared to control. In addition, there were no significant changes in HOMA-IR in the L-HIIT (MD 0.51 (−0.11 to 1.12); *p* = 0.1) or H-HIIT (MD −0.12 (−0.73 to 0.48); *p* = 0.68) groups after the intervention period compared to control (Supplementary Table 1).

## Discussion

This study has shown that just 4 weeks, time-efficient, equipment-free, bodyweight-based HIIT is able to elicit improvements in the CRF and BMI of young healthy adults, and also aspects of skeletal muscle architecture, all irrespective of exercise supervision. This HIIT protocol was not however able to elicit improvements in resting BP, or indices of glucose and insulin metabolism in this cohort.

Although HIIT has been well-documented to be a potent stimulus to improve CRF in a short period of time in younger adults (Gibala et al., 2012; Gillen and Gibala, 2014; MacInnis and Gibala, 2017), the role of supervision and the requirement for equipment to facilitate efficacious HIIT in this age group has been scarcely studied (Blackwell et al., 2017; Scott et al., 2019). This study addresses this evidence gap and demonstrates that: (1) HIIT is acceptable and effective for young individuals in a non-laboratory environment; (2) bodyweight-based HIIT can achieve comparable improvements in CRF to those achieved using equipment (e.g., cycle ergometers); and (3) these improvements can be achieved using an unsupervised protocol with no direct feedback. The improvements we report in both our L-HIIT and H-HIIT groups provides evidence that the exercises employed were sufficient to generate a high enough intensity to replicate the previously reported benefits of HIIT with equipment (Boereboom et al., 2016; MacInnis and Gibala, 2017); with exercise intensity shown to be key to achieving improvements in CRF (Phillips et al., 2017). Our HIIT programme was well-received by all participants and no adverse events or injuries were reported during completion of the programme, irrespective of supervision. This data supports the growing notion that HIIT can be performed outside a laboratory/supervised environment, and that expensive specialist equipment is not needed (Hardcastle et al., 2014; Metcalfe et al., 2020).

Higher fitness levels at an earlier age are associated with a lower future risk for all-cause mortality and adverse cardiovascular events (Valle et al., 2015; Shah et al., 2016). As such, the significant improvements CRF that we report after just 4 weeks of intervention provides further evidence that HIIT as an independent exercise modality potentially mitigates future disease risk. Compared to health-based physical activity guidelines, which until recently have primarily focussed on moderate intensity continuous training (MICT), the ~10% increase in CRF that we report for both of our HIIT groups is consistent with those previously observed with MICT [e.g., 11% after 5-weeks cycling (Kong et al., 2016)].

It must be recognised that HIIT is an umbrella term used for numerous different exercise profiles which intersperse intense efforts with periods of rest or active recovery (Gibala and Little, 2010). Some of these protocols are not time-efficient and as such do not address the commonly cited barrier to exercise of “lack of time.” Conversely, some HIIT protocols are incredibly time-efficient (Whyte et al., 2010) but require “supra-maximal” efforts [often known as sprint-interval training (SIT)], which are often considered not suitable, or indeed attainable, by those with no prior exercise training experience (Gillen and Gibala, 2014). Using a temporal HIIT protocol which is both time-efficient (<16 min per session) and achievable by all [including cancer patients (Blackwell et al., 2020) and elderly individuals (Boereboom et al., 2016)], translated into an equipment free regime, our results are superior to that observed in a 6-week, cycle ergometer-based HIIT study which employed a four-by-30 s protocol in overweight or obese young males. This study reported only a 2.8% increase in VO_2_peak (Fisher et al., 2015) and concluded that 6 weeks of MICT using continuous cycling (+11.1%) was superior to HIIT for improving CRF in this group. Although between group differences (BMI and gender split) may explain the discrepant findings between the work of Fisher and colleagues and those presented herein, we postulate that it was likely the duration of high-intensity time (2 vs. 5 min) per session, as session frequency and target intensity were matched.

The findings from this study are comparable to another bodyweight based HIIT study in young, obese individuals, which at 4 weeks post intervention, reported an improvement in VO_2_peak of 2.6 ml/kg/min (Scott et al., 2019). This non-randomised study utilised a four-by-60 s protocol performed 3 times per week for 12 weeks, with interim results at 4 weeks. That our results are similar to this study further validates the use of a 60 s effort for bodyweight HIIT protocols to elicit improvements in CRF.

The findings from the HIIT regime employed in our study are also superior to the findings of a meta-analysis of <13 sessions of low volume HIIT which reported a 6.2% and 3.6% improvement in the VO_2_peak of young, active, non-athletic males and females, respectively (Weston M. et al., 2014). The majority of studies in this meta-analysis were fully supervised and had equipment requirements. As such, that the improvements we report in this study were based on a protocol with no equipment and a much-reduced time commitment when compared to both MICT and some other forms of HIIT, our HIIT regimen may also improve adherence given that both equipment and time are commonly cited barriers to exercise (Foster et al., 2015).

An increase of one MET or 3.5 ml/kg/min (Jetté et al., 1990) in VO_2_peak is associated with a 15 and 19% lower risk of all-cause and CVD mortality, respectively (Lee et al., 2011). The increase in VO_2_peak that we observed in both HIIT groups is close to this level and not different based on supervision, supporting the notion of this HIIT protocol being a feasible strategy for reducing the risk of all-cause mortality in later life. However, for this benefit to be achieved exercise adherence would be essential, and as such further large-scale trials are needed to not only assess exercise adherence but also retention of favourable adaptations beyond the 4-week intervention period reported in this study.

BMI is another parameter known to be closely associated with cardio-metabolic disease risk (Isomaa et al., 2001; Kassi et al., 2011) and we report small but significant reductions in BMI in both HIIT groups, representing another favourable physiological impact of our HIIT regime. These relatively small improvements in BMI in our non-obese cohort are similar to those reported in a 12-week rowing HIIT study in non-obese young females (Brown et al., 2018). Further, that an 8-week SIT programme comparing improvements in BMI and body composition in young obese and normal weight males reported significant improvements in the obese group only (Ouerghi et al., 2017), suggests that greater improvements in BMI may be observed using our HIIT regime in individuals with a less favourable body composition profile at baseline.

A second aspect of body composition associated with overall health is skeletal muscle mass (Franchi et al., 2018). In this study, although we were not able to assess whole-body muscle mass as reported in a number of HIIT studies (Boereboom et al., 2016; Brown et al., 2018; Marcos-Pardo et al., 2019; Scott et al., 2019), we saw no change in VL MT, reported to be representative of muscle mass (Franchi et al., 2018) or VL FL (Randhawa and Wakeling, 2015) in any of the groups. We did however observe an increase in PA in both HIIT groups which is thought to represent an increase in cross sectional area and associated force and contractile capability (Lee et al., 2015; Monti et al., 2020). Whilst improvements in muscle PA have been shown following training modalities commonly associated with hypertrophy and strength gains, e.g., resistance and plyometric exercise training (Monti et al., 2020), the impact of HIIT on PA has been less well-studied.

Despite many HIIT studies reporting improved blood glucose and insulin sensitivity in healthy young, adults after training (Ciolac et al., 2010; Fisher et al., 2015; Shepherd et al., 2015), and in those with impaired metabolic function (Whyte et al., 2010; Jelleyman et al., 2015; Scott et al., 2019), we did not demonstrate any significant improvements in indices of glucose or insulin metabolism, each key features of whole-body health and future disease risk. Of these previous studies in healthy young adults, the shortest training duration was 6-weeks and as such it may be that the 4-week protocol employed in this study was too short to elicit improvements in indices of glucose handling and insulin sensitivity. However, somewhat against the suggestion of training duration being the limiting factor for improvements in these parameters, Babraj et al. (2009) showed improvements in glucose and insulin metabolism in healthy young males following just 2 weeks of a supervised, cycle-based SIT protocol. However, as already mentioned, the “supra-maximal” style of SIT would be difficult to achieve outside of a laboratory setting, especially without equipment, and may not appeal to many individuals (Gillen and Gibala, 2014). As such it may be that a longer duration of training is needed for our well-tolerated, HIIT protocol to elicit favourable changes in glucose and insulin metabolism, especially in young healthy individuals without metabolic impairment.

A number of studies have shown HIIT to be an excellent way to improve BP in distinct patient cohorts, and young and older adults (Ciolac et al., 2010; Gillen et al., 2014; Bruseghini et al., 2015; Grace et al., 2018), including those using the same temporal HIIT profile (Herrod et al., 2020) and/or training duration reported herein (Boereboom et al., 2016). However, in this study, we failed to demonstrate any change in resting SBP or DBP in either HIIT group. We are not however the first to report a lack of improvement in BP with HIIT. For example, a 6 week cycle ergometer-based study in young, overweight males also failed to demonstrate improvements (Fisher et al., 2015). As with our suggestion for improvements in metabolic function, it may be that in normotensive, younger participants, a longer duration of HIIT is required to elicit changes in BP, especially via our well-tolerated regime. Furthermore, a separate study in young, overweight males performing 6-weeks of cycle ergometer-based HIIT reported that BP reductions were less pronounced in normotensive subjects (Clark et al., 2020), which may go some way to explain our findings.

Self-monitoring has been shown to be a promising technique to invoke positive behavioural changes in physical inactivity, thereby increasing rates of compliance and adherence to exercise interventions (Gardner et al., 2016). As such, we must consider that our request for self-report in the H-HIIT group may have impacted their behaviour. In addition, more modern eHealth technologies have been shown to reduce the burden and improve the accuracy of physical activity data collection, when compared to traditional paper-based methods (Compernolle et al., 2019). Further, these technologies have also been shown to improve compliance, adherence and in turn greater achievement of intervention goals (Turner-McGrievy et al., 2013). Although self-reported levels of adherence and compliance in our H-HIIT group were high, the use of eHealth technologies with the opportunity to integrate assessment and feedback, may further enhance motivation and adherence/compliance, especially over longer periods of training. For example, in a recent unsupervised, equipment-free HIIT study, Scott et al. (2019) reported improvements in cardiometabolic health and good levels of adherence in younger adults with diabetes completing a 12-week protocol. Scott and colleagues monitored HR remotely with feedback and instruction provided to participants via a mobile phone application (Scott et al., 2019). Whilst unsupervised HIIT has not been extensively investigated, the application of HIIT in this way may prove to be a crucial cost-effective public health tool to battle the rise of inactivity, cardiometabolic disorders and the associated later poor health outcomes in younger adults.

As with all studies, we recognise some limitations to this work. Firstly, as a volunteer study, self-selection to take part may have created bias within our final cohort. Although this would not impact between-group comparisons, or indeed conclusions on the efficacy of our intervention, it may be that compliance and adherence data may not represent that to be achieved by a larger scale public-health “roll-out.” More expansive measures of body composition would provide additional information on other aspects pertaining to whole body health that may be achieved by our HIIT regimen [i.e., adipose distribution (Scott et al., 2019) and or bone facets (Ravnholt et al., 2018)]. The use of a heart rate monitor, ideally with remote monitoring capability, would have improved the reliability of the data on heart rate achieved during each session/effort, and this is scope for future work. Whilst participants were asked to maintain their current level of physical activity for the duration of the study, this was not measured, and as such it cannot be concluded that alterations in this aspect of their lifestyle did not influence outcomes of this study. Finally, although the inclusion of males and females is a strength of this study given its potential application for public health, hormonal changes during the menstrual cycle are known to impact exercise adaptation and performance (Janse de Jonge, 2003). Despite this, numbers of females in each group was roughly equal, and the 4-week training duration would span a regular menstrual cycle in those not take contraceptive medication, and as such we do not feel that not controlling for menstrual cycle phase will have likely impacted our outcomes.

## Conclusion

A 4-week, time-efficient, equipment-free HIIT programme is able to elicit improvements in the CRF and BMI of young, healthy adults. Furthermore, this programme is equally successful when performed in an unsupervised setting such as the home. Our home-based HIIT regime could offer an attractive exercise intervention for younger adults, counteracting some of the effects of the rise in sedentary behaviour, and potentially mitigating chronic disease risk across the adult life-course.

## Data Availability Statement

The raw data supporting the conclusions of this article will be made available by the authors, without undue reservation.

## Ethics Statement

The studies involving human participants were reviewed and approved by University of Nottingham Faculty of Medicine and Health Sciences Research Ethics Committee (C16122016). The patients/participants provided their written informed consent to participate in this study.

## Author Contributions

TS, JL, and BP: study conception and design. TS, TI, and AG: acquisition of data. TS, BD, NG, PA, and BP: analysis and interpretation of data. TS, PA, and BP: wrote manuscript. All authors contributed to the final approval of manuscript.

## Conflict of Interest

The authors declare that the research was conducted in the absence of any commercial or financial relationships that could be construed as a potential conflict of interest.

## Publisher's Note

All claims expressed in this article are solely those of the authors and do not necessarily represent those of their affiliated organizations, or those of the publisher, the editors and the reviewers. Any product that may be evaluated in this article, or claim that may be made by its manufacturer, is not guaranteed or endorsed by the publisher.

## References

[B1] Babraj J. A. Vollaard N. B. Keast C. Guppy F. M. Cottrell G. Timmons J. A. (2009). Extremely short duration high intensity interval training substantially improves insulin action in young healthy males. BMC Endocr. Disord. 9:3. 10.1186/1472-6823-9-3 19175906 PMC2640399

[B2] Blackwell J. Atherton P. J. Smith K. Doleman B. Williams J. P. Lund J. N.. (2017). The efficacy of unsupervised home-based exercise regimens in comparison to supervised laboratory-based exercise training upon cardio-respiratory health facets. Physiol. Rep. 5:13390. 10.14814/phy2.13390 28912129 PMC5599857

[B3] Blackwell J. E. M. Doleman B. Boereboom C. L. Morton A. Williams S. Atherton P.. (2020). High-intensity interval training produces a significant improvement in fitness in less than 31 days before surgery for urological cancer: a randomised control trial. Prostate Cancer Prostatic Dis. 23, 696–704. 10.1038/s41391-020-0219-1 32157250 PMC7655502

[B4] Boereboom C. Phillips B. E. Williams J. P. Lund J. N. (2016). A 31-day time to surgery compliant exercise training programme improves aerobic health in the elderly. Tech. Coloproctol. 20, 375–382. 10.1007/s10151-016-1455-1 27015678

[B5] Booth F. W. Roberts C. K. Thyfault J. P. Ruegsegger G. N. Toedebusch R. G. (2017). Role of inactivity in chronic diseases: evolutionary insight and pathophysiological mechanisms. Physiol. Rev. 97, 1351–1402. 10.1152/physrev.00019.2016 28814614 PMC6347102

[B6] Brown E. C. Hew-Butler T. Marks C. R. C. Butcher S. J. Choi M. D. (2018). The impact of different high-intensity interval training protocols on body composition and physical fitness in healthy young adult females. Biores. Open Access 7, 177–185. 10.1089/biores.2018.0032 30622842 PMC6323591

[B7] Bruseghini P. Calabria E. Tam E. Milanese C. Oliboni E. Pezzato A.. (2015). Effects of eight weeks of aerobic interval training and of isoinertial resistance training on risk factors of cardiometabolic diseases and exercise capacity in healthy elderly subjects. Oncotarget 6:16998. 10.18632/oncotarget.4031 26046575 PMC4627287

[B8] Carnethon M. R. Gidding S. S. Nehgme R. Sidney S. Jacobs D. R. Jr Liu K. (2003). Cardiorespiratory fitness in young adulthood and the development of cardiovascular disease risk factors. JAMA 290:3092. 10.1001/jama.290.23.3092 14679272

[B9] Castro O. Bennie J. Vergeer I. Bosselut G. Biddle S. J. H. (2018). Correlates of sedentary behaviour in university students: a systematic review. Prev. Med. 116, 194–202. 10.1016/j.ypmed.2018.09.016 30266213

[B10] Chow L. Odegaard A. Bosch T. Bantle A. Wang Q. Hughes J.. (2016). Twenty year fitness trends in young adults and incidence of prediabetes and diabetes: the CARDIA study. Diabetologia 59, 1659–1665. 10.1007/s00125-016-3969-5 27181604 PMC4930716

[B11] Ciolac E. G. Bocchi E. A. Bortolotto L. A. Carvalho V. O. Greve J. M. Guimarães G. V. (2010). Effects of high-intensity aerobic interval training vs. moderate exercise on hemodynamic, metabolic and neuro-humoral abnormalities of young normotensive women at high familial risk for hypertension. Hypertens. Res. 33, 836–843. 10.1038/hr.2010.72 20448634

[B12] Clark T. Morey R. Jones M. D. Marcos L. Ristov M. Ram A.. (2020). High-intensity interval training for reducing blood pressure: a randomized trial vs. moderate-intensity continuous training in males with overweight or obesity. Hypertens. Res. 43, 396–403. 10.1038/s41440-019-0392-6 31937915

[B13] Compernolle S. DeSmet A. Poppe L. Crombez G. De Bourdeaudhuij I. Cardon G.. (2019). Effectiveness of interventions using self-monitoring to reduce sedentary behavior in adults: a systematic review and meta-analysis. Int. J. Behav. Nutr. Phys. Act. 16:63. 10.1186/s12966-019-0824-3 31409357 PMC6693254

[B14] de Boer M. R. Waterlander W. E. Kuijper L. D. J. Steenhuis I. H. M. Twisk J. W. R. (2015). Testing for baseline differences in randomized controlled trials: an unhealthy research behavior that is hard to eradicate. Int. J. Behav. Nutr. Phys. Act. 12:4. 10.1186/s12966-015-0162-z 25616598 PMC4310023

[B15] Ekelund U. Steene-Johannessen J. Brown W. J. Fagerland M. W. Owen N. Powell K. E.. (2016). Does physical activity attenuate, or even eliminate, the detrimental association of sitting time with mortality? A harmonised meta-analysis of data from more than 1 million men and women. Lancet 388, 1302–1310. 10.1016/S0140-6736(16)30370-1 27475271

[B16] Fisher G. Brown A. W. Bohan Brown M. M. Alcorn A. Noles C. Winwood L.. (2015). High intensity interval- vs moderate intensity- training for improving cardiometabolic health in overweight or obese males: a randomized controlled trial. PLoS ONE 10:e0138853. 10.1371/journal.pone.0138853 26489022 PMC4619258

[B17] Foster C. Farland C. V. Guidotti F. Harbin M. Roberts B. Schuette J.. (2015). The effects of high intensity interval training vs steady state training on aerobic and anaerobic capacity. J. Sports Sci. Med. 14:747. 10.1249/01.mss.0000476771.63318.52 26664271 PMC4657417

[B18] Fox S. M. Naughton J. P. Haskell W. L. (1971). Physical activity and the prevention of coronary heart disease. Ann. Clin. Res. 3, 404–432. 4945367

[B19] Franchi M. V. Longo S. Mallinson J. Quinlan J. I. Taylor T. Greenhaff P. L.. (2018). Muscle thickness correlates to muscle cross-sectional area in the assessment of strength training-induced hypertrophy. Scand. J. Med. Sci. Sports 28, 846–853. 10.1111/sms.12961 28805932 PMC5873262

[B20] Gardner B. Smith L. Lorencatto F. Hamer M. Biddle S. J. (2016). How to reduce sitting time? A review of behaviour change strategies used in sedentary behaviour reduction interventions among adults. Health Psychol. Rev. 10, 89–112. 10.1080/17437199.2015.1082146 26315814 PMC4743603

[B21] Gibala M. J. Little J. P. (2010). Just HIT it! A time-efficient exercise strategy to improve muscle insulin sensitivity. J. Physiol. 588, 3341–3342. 10.1113/jphysiol.2010.196303 20843832 PMC2988497

[B22] Gibala M. J. Little J. P. Macdonald M. J. Hawley J. A. (2012). Physiological adaptations to low-volume, high-intensity interval training in health and disease. J. Physiol. 590, 1077–1084. 10.1113/jphysiol.2011.224725 22289907 PMC3381816

[B23] Gillen J. B. Gibala M. J. (2014). Is high-intensity interval training a time-efficient exercise strategy to improve health and fitness? Appl. Physiol. Nutr. Metab. 39, 409–412. 10.1139/apnm-2013-0187 24552392

[B24] Gillen J. B. Percival M. E. Skelly L. E. Martin B. J. Tan R. B. Tarnopolsky M. A.. (2014). Three minutes of all-out intermittent exercise per week increases skeletal muscle oxidative capacity and improves cardiometabolic health. PLoS ONE 9:e111489. 10.1371/journal.pone.0111489 25365337 PMC4218754

[B25] Grace F. Herbert P. Elliott A. D. Richards J. Beaumont A. Sculthorpe N. F. (2018). High intensity interval training (HIIT) improves resting blood pressure, metabolic (MET) capacity and heart rate reserve without compromising cardiac function in sedentary aging men. Exp. Gerontol. 109, 75–81. 10.1016/j.exger.2017.05.010 28511954

[B26] Guthold R. Stevens G. A. Riley L. M. Bull F. C. (2020). Global trends in insufficient physical activity among adolescents: a pooled analysis of 298 population-based surveys with 1·6 million participants. Lancet Child Adolesc. Heal. 4, 23–35. 10.1016/S2352-4642(19)30323-2 31761562 PMC6919336

[B27] Hardcastle S. J. Ray H. Beale L. Hagger M. S. (2014). Why sprint interval training is inappropriate for a largely sedentary population. Front. Psychol. 5:1505. 10.3389/fpsyg.2014.01505 25566166 PMC4274872

[B28] Herrod P. J. J. Blackwell J. E. M. Boereboom C. L. Atherton P. J. Williams J. P. Lund J. N.. (2020). The time course of physiological adaptations to high-intensity interval training in older adults. Aging Med. 3, 245–251. 10.1002/agm2.12127 33392430 PMC7771560

[B29] Hwang J. Kim K. Brothers R. M. Castelli D. M. Gonzalez-Lima F. (2018). Association between aerobic fitness and cerebrovascular function with neurocognitive functions in healthy, young adults. Exp. Brain Res. 236, 1421–1430. 10.1007/s00221-018-5230-6 29536150

[B30] Isomaa B. Almgren P. Tuomi T. Forsén B. Lahti K. Nissén M.. (2001). Cardiovascular morbidity and mortality associated with the metabolic syndrome. Diabetes Care 24, 683–689. 10.2337/diacare.24.4.683 11315831

[B31] Janse de Jonge X. A. K. (2003). Effects of the menstrual cycle on exercise performance. Sport Med. 33, 833–851. 10.2165/00007256-200333110-00004 12959622

[B32] Jelleyman C. Yates T. O'Donovan G. Gray L. J. King J. A. Khunti K.. (2015). The effects of high-intensity interval training on glucose regulation and insulin resistance: a meta-analysis. Obes. Rev. 16, 942–961. 10.1111/obr.12317 26481101

[B33] Jetté M. Sidney K. Blümchen G. (1990). Metabolic equivalents (METS) in exercise testing, exercise prescription, and evaluation of functional capacity. Clin. Cardiol. 13, 555–565. 10.1002/clc.4960130809 2204507

[B34] Kaczmarek S. Habedank D. Obst A. Dörr M. Völzke H. Gläser S.. (2019). Interobserver variability of ventilatory anaerobic threshold in asymptomatic volunteers. Multidiscip. Respir. Med. 14:20. 10.4081/mrm.2019.21 31198557 PMC6556958

[B35] Karlsen T. Aamot I.-L. Haykowsky M. Rognmo Ø. (2017). High intensity interval training for maximizing health outcomes. Prog. Cardiovasc. Dis. 60, 67–77. 10.1016/j.pcad.2017.03.006 28385556

[B36] Kassi E. Pervanidou P. Kaltsas G. Chrousos G. (2011). Metabolic syndrome: definitions and controversies. BMC Med. 9:48. 10.1186/1741-7015-9-48 21542944 PMC3115896

[B37] Kodama S. Saito K. Tanaka S. Maki M. Yachi Y. Asumi M.. (2009). Cardiorespiratory fitness as a quantitative predictor of all-cause mortality and cardiovascular events in healthy men and women. JAMA 301:2024. 10.1001/jama.2009.681 19454641

[B38] Kong Z. Sun S. Liu M. Shi Q. (2016). Short-term high-intensity interval training on body composition and blood glucose in overweight and obese young women. J. Diabetes Res. 2016:4073618. 10.1155/2016/4073618 27774458 PMC5059579

[B39] Korkiakangas E. E. Alahuhta M. A. Husman P. M. Keinänen-Kiukaanniemi S. Taanila A. M. Laitinen J. H. (2011). Motivators and barriers to exercise among adults with a high risk of type 2 diabetes - a qualitative study. Scand. J. Caring Sci. 25, 62–69. 10.1111/j.1471-6712.2010.00791.x 20384973

[B40] Lee D. Li Z. Sohail Q. Z. Jackson K. Fiume E. Agur A. (2015). A three-dimensional approach to pennation angle estimation for human skeletal muscle. Comput. Methods Biomech. Biomed. Eng. 18, 1474–1484. 10.1080/10255842.2014.917294 24849037

[B41] Lee D. Sui X. Artero E. G. Lee I.-M. Church T. S. McAuley P. A.. (2011). Long-term effects of changes in cardiorespiratory fitness and body mass index on all-cause and cardiovascular disease mortality in men: the Aerobics Center Longitudinal Study. Circulation 124, 2483–2490. 10.1161/CIRCULATIONAHA.111.038422 22144631 PMC3238382

[B42] Levett D. Z. H. Jack S. Swart M. Carlisle J. Wilson J. Snowden C.. (2018). Perioperative cardiopulmonary exercise testing (CPET): consensus clinical guidelines on indications, organization, conduct, and physiological interpretation. Br. J. Anaesth. 120, 484–500. 10.1016/j.bja.2017.10.020 29452805

[B43] MacInnis M. J. Gibala M. J. (2017). Physiological adaptations to interval training and the role of exercise intensity. J. Physiol. 595:2915. 10.1113/JP273196 27748956 PMC5407969

[B44] Magnussen C. G. Koskinen J. Chen W. Thomson R. Schmidt M. D. Srinivasan S. R.. (2010). Pediatric metabolic syndrome predicts adulthood metabolic syndrome, subclinical atherosclerosis, and type 2 diabetes mellitus but is no better than body mass index alone: the Bogalusa Heart Study and the Cardiovascular Risk in Young Finns Study. Circulation 122, 1604–1611. 10.1161/CIRCULATIONAHA.110.940809 20921439 PMC3388503

[B45] Marcos-Pardo P. J. Orquin-Castrillón F. J. Gea-García G. M. Menayo-Antúnez R. González-Gálvez N. Vale R.. (2019). Effects of a moderate-to-high intensity resistance circuit training on fat mass, functional capacity, muscular strength, and quality of life in elderly: a randomized controlled trial. Sci. Rep. 9:7830. 10.1038/s41598-019-44329-6 31127163 PMC6534570

[B46] Metcalfe R. S. Atef H. Mackintosh K. McNarry M. Ryde G. Hill D. M.. (2020). Time-efficient and computer-guided sprint interval exercise training for improving health in the workplace: a randomised mixed-methods feasibility study in office-based employees. BMC Public Health 20:313. 10.1186/s12889-020-8444-z 32164631 PMC7068982

[B47] Monti E. Franchi M. V. Badiali F. Quinlan J. I. Longo S. Narici M. V. (2020). The time-course of changes in muscle mass, architecture and power during 6 weeks of plyometric training. Front. Physiol. 11:946. 10.3389/fphys.2020.00946 32848873 PMC7417646

[B48] Ouerghi N. Fradj M. K. Ben Bezrati I. Khammassi M. Feki M. Kaabachi N.. (2017). Effects of high-intensity interval training on body composition, aerobic and anaerobic performance and plasma lipids in overweight/obese and normal-weight young men. Biol. Sport 34, 385–392. 10.5114/biolsport.2017.69827 29472742 PMC5819474

[B49] Owen N. Healy G. N. Matthews C. E. Dunstan D. W. (2010). Too much sitting: the population health science of sedentary behavior. Exerc. Sport Sci. Rev. 38, 105–103. 10.1097/JES.0b013e3181e373a2 20577058 PMC3404815

[B50] Patterson R. McNamara E. Tainio M. de Sá T. H. Smith A. D. Sharp S. J.. (2018). Sedentary behaviour and risk of all-cause, cardiovascular and cancer mortality, and incident type 2 diabetes: a systematic review and dose response meta-analysis. Eur. J. Epidemiol. 33, 811–829. 10.1007/s10654-018-0380-1 29589226 PMC6133005

[B51] Phillips B. Kelly B. Lilja M. Ponce-González J. G. Brogan R. J. Morris D. L.. (2017). A practical and time-efficient high-intensity interval training program modifies cardio-metabolic risk factors in adults with risk factors for type II diabetes. Front. Endocrinol. 8:229. 10.3389/fendo.2017.00229 28943861 PMC5596071

[B52] Randhawa A. Wakeling J. M. (2015). Multidimensional models for predicting muscle structure and fascicle pennation. J. Theor. Biol. 382, 57–63. 10.1016/j.jtbi.2015.06.001 26073723

[B53] Ravnholt T. Tybirk J. Jørgensen N. R. Bangsbo J. (2018). High-intensity intermittent “5-10-15” running reduces body fat, and increases lean body mass, bone mineral density, and performance in untrained subjects. Eur. J. Appl. Physiol. 118, 1221–1230. 10.1007/s00421-018-3851-x 29594355 PMC5966499

[B54] Rueden C. T. Schindelin J. Hiner M. C. DeZonia B. E. Walter A. E. Arena E. T.. (2017). ImageJ2: ImageJ for the next generation of scientific image data. BMC Bioinform. 18:529. 10.1186/s12859-017-1934-z 29187165 PMC5708080

[B55] Scott S. N. Shepherd S. O. Hopkins N. Dawson E. A. Strauss J. A. Wright D. J.. (2019). Home-hit improves muscle capillarisation and eNOS/NAD(P)Hoxidase protein ratio in obese individuals with elevated cardiovascular disease risk. J. Physiol. 597, 4203–4225. 10.1113/JP278062 31218680

[B56] Scott S. N. Shepherd S. O. Strauss J. A. Wagenmakers A. J. M. Cocks M. (2020). Home-based high-intensity interval training reduces barriers to exercise in people with type 1 diabetes. Exp. Physiol. 105, 571–578. 10.1113/EP088097 31584734

[B57] Shah R. V. Murthy V. L. Colangelo L. A. Reis J. Bharath Ambale Venkatesh M. Sharma R.. (2016). Association of fitness in young adulthood with survival and cardiovascular risk the coronary artery risk development in young adults (CARDIA) study HHS public access. JAMA Intern. Med. 176, 87–95. 10.1001/jamainternmed.2015.6309 26618471 PMC5292201

[B58] Shepherd S. O. Wilson O. J. Taylor A. S. Thøgersen-Ntoumani C. Adlan A. M. Wagenmakers A. J. M.. (2015). Low-volume high-intensity interval training in a gym setting improves cardio-metabolic and psychological health. PLoS ONE 10:e0139056. 10.1371/journal.pone.0139056 26402859 PMC4581708

[B59] Sparling P. B. Howard B. J. Dunstan D. W. Owen N. (2015). Recommendations for physical activity in older adults. BMJ 350:h100. 10.1136/bmj.h100 25608694

[B60] Tremblay M. S. Aubert S. Barnes J. D. Saunders T. J. Carson V. Latimer-Cheung A. E.. (2017). Sedentary Behavior Research Network (SBRN) – terminology consensus project process and outcome. Int. J. Behav. Nutr. Phys. Act. 14:75. 10.1186/s12966-017-0525-8 28599680 PMC5466781

[B61] Turner-McGrievy G. M. Beets M. W. Moore J. B. Kaczynski A. T. Barr-Anderson D. J. Tate D. F. (2013). Comparison of traditional versus mobile app self-monitoring of physical activity and dietary intake among overweight adults participating in an mHealth weight loss program. J. Am. Med. Inform. Assoc. 20, 513–518. 10.1136/amiajnl-2012-001510 23429637 PMC3628067

[B62] Vainshelboim B. Brennan G. M. LoRusso S. Fitzgerald P. Wisniewski K. S. (2019). Sedentary behavior and physiological health determinants in male and female college students. Physiol. Behav. 204, 277–282. 10.1016/j.physbeh.2019.02.041 30831185

[B63] Valle C. G. Tate D. F. Mayer D. K. Allicock M. Cai J. Campbell M. K. (2015). Physical activity in young adults: a signal detection analysis of Health Information National Trends Survey (HINTS) 2007 data. J. Health Commun. 20, 134–146. 10.1080/10810730.2014.917745 25375396 PMC4852744

[B64] Weisman I. M. Marciniuk D. Martinez F. J. Sciurba F. Sue D. Myers Bruce Johnson J.. (2003). ATS/ACCP statement on cardiopulmonary exercise testing. Am. J. Respir. Crit. Care Med. 167, 211–277. 10.1164/ajrccm.167.10.952 12524257

[B65] Weston K. S. Wisløff U. Coombes J. S. (2014). High-intensity interval training in patients with lifestyle-induced cardiometabolic disease: a systematic review and meta-analysis. Br. J. Sports Med. 48, 1227–1234. 10.1136/bjsports-2013-092576 24144531

[B66] Weston M. Taylor K. L. Batterham A. M. Hopkins W. G. (2014). Effects of low-volume high-intensity interval training (HIT) on fitness in adults: a meta-analysis of controlled and non-controlled trials. Sport. Med. 44, 1005–1017. 10.1007/s40279-014-0180-z 24743927 PMC4072920

[B67] Whyte L. J. Gill J. M. R. Cathcart A. J. (2010). Effect of 2 weeks of sprint interval training on health-related outcomes in sedentary overweight/obese men. Metab. Clin. Exp. 59, 1421–1428. 10.1016/j.metabol.2010.01.002 20153487

[B68] Wilmot E. G. Edwardson C. L. Achana F. A. Davies M. J. Gorely T. Gray L. J.. (2012). Sedentary time in adults and the association with diabetes, cardiovascular disease and death: systematic review and meta-analysis. Diabetologia 55, 2895–2905. 10.1007/s00125-012-2677-z 22890825

[B69] World Health Organisation (WHO). (2006). Definition and Diagnosis of Diabetes Mellitus and Intermediate Hyperglycemia: Report of a WHO/IDF ConsultatIon. International Diabetets Federation (IDF), WHO, Geneva, Switzerland.

[B70] Young D. R. Hivert M.-F. Alhassan S. Camhi S. M. Ferguson J. F. Katzmarzyk P. T.. (2016). Sedentary behavior and cardiovascular morbidity and mortality: a science advisory from the American Heart Association. Circulation 134:e262–e279. 10.1161/CIR.0000000000000440 27528691

