# Supplementary material for: Short-Term, Equipment-Free High Intensity Interval Training Elicits Significant Improvements in Cardiorespiratory Fitness Irrespective of Supervision in Early Adulthood
Source: Front Sports Act Living. 2021 Jul 26;3:697518. doi: 10.3389/fspor.2021.697518 (PMC8349982; doi:10.3389/fspor.2021.697518)
Supplement: Supplementary file 1 [file Table_1.docx]

**Supplementary Information**

|  | **L-HIIT** | | **H-HIIT** | | **CON** | |
| --- | --- | --- | --- | --- | --- | --- |
|  | **Pre** | **Post** | **Pre** | **Post** | **Pre** | **Post** |
| **AT**  **(ml/kg/min)** | 14.89 (4.6) | 17.09 (4.6)** | 20.27 (5.8) | 22.63 (5.4)** | 18.22 (4.2) | 17.86 (3.8) |
| **VO_2_peak**  **(ml/kg/min)** | 31.08 (7.9) | 34.57 (8.4)** | 36.06 (4.8) | 39.19 (4.8)** | 37.86 (8.8) | 38.35 (9.0) |
| **SBP**  **(mmHg)** | 120 (11) | 117 (9) | 126 (6) | 123 (8) | 118 (14) | 118 (12) |
| **DBP**  **(mmHg)** | 74 (7) | 71 (6) | 76 (8) | 75 (7) | 73 (7) | 73 (11) |
| **BMI**  **(kg/m^2^)** | 24.59 (4) | 24.4 (4)** | 25.54 (4) | 25.23 (4)* | 24.9 (4) | 24.9 (4) |
| **PA**  **(degrees)** | 14.9 (3) | 17.8 (2)** | 13.1 (5) | 15.3 (6)** | 15.1 (2) | 14.7 (1) |
| **MT**  **(mm)** | 2.36 (0.4) | 2.43 (0.4) | 2.31 (0.9) | 2.30 (0.9) | 2.49 (0.5) | 2.53 (0.6) |
| **FL**  **(mm)** | 7.18 (2) | 7.08 (2) | 6.93 (3) | 7.17 (3) | 6.94 (2) | 6.97 (2) |
| **Glucose AUC** | 819.4 (110) | 785 (102) | 796 (159) | 776.4 (155) | 764.9 (122) | 816.1 (117) |
| **Insulin**  **AUC** | 5445.4 (2421) | 6914.4  (3769) | 8154.8 (5145) | 7525.9 (2698) | 6330.4 (3392) | 6193.4 (2233) |
| **HOMA-IR** | 1.1 (0.7) | 1.6 (1.0) | 1.6 (0.7) | 1.3 (0.5) | 1.4 (0.7) | 1.3 (0.5) |

**Supplementary Table 1 (S1):** Assessment parameters before (pre) and after (post) a 4-week period of L-HIIT, H-HIIT or a CON period. Abbreviations: L-HIIT, Laboratory (supervised) high intensity interval training; H-HIIT, Home-based (unsupervised) HIIT; CON, no-intervention control group; AT, anaerobic threshold; SBP, systolic blood pressure; DBP, diastolic blood pressure; BMI, body mass index; PA, (muscle) pennation angle; MT, muscle thickness; FL, (muscle) fascicle length); AUC, area under the curve (presented as mmol/120 minutes for glucose and uU/ml/120 minutes for insulin); HOMA-IR, homeostatic model assessment of insulin resistance. Data are presented as mean (SD). Analysis via repeated measures t-tests. *=p<0.05, **=p<0.01 versus pre-intervention in the same group.
